# Synchronous Perforation of the Ileum and Meckel’s Diverticulum Due to Tuberculosis

**DOI:** 10.4021/gr2010.04.181e

**Published:** 2010-03-20

**Authors:** Sanoop K. Zachariah

**Affiliations:** Department of Surgery, MOSC Medical College, Kolenchery, Cochin, India. Email: skzach@yahoo.com

**Keywords:** Meckel's diverticulum, Intestinal tuberculosis, Ileal perforation

## Abstract

Perforation of the Meckel’s diverticulum due to tuberculosis is a rare phenomenon. A 45 years old male who presented with perforation peritonitis was found to have a synchronous dual perforation involving the ileum and the Meckel’s diverticulum, due to intestinal tuberculosis. In addition to this, the Meckel’s diverticulum was found to have a daughter diverticulum (diverticulum within diverticulum), which was probably pulsion or traction diverticulum as it did not have all layers of the intestinal wall. Such daughter diverticulum associated with a Meckel’s diverticulum is very unusual. All these factors make this a unique case which is hence reported here.

## Introduction

Meckel’s diverticulum (MD) is the commonest congenital gastrointestinal anomaly. It was first described in detail by Hohann Friedrick Meckel in 1808 and thus bears his name [1]. It is a true diverticulum, consisting of all intestinal layers and is due to the persistence of the vitellointestinal duct. It is present in approximately 2% percent of the population with a male : female ratio of 2 : 1 and approximately 20% may contain ectopic gastric mucosa [2]. A rare case of tuberculous dual perforation of the ileum along with the Meckel’s diverticulum in an adult is presented here.

## Case Report

A 45 years old male presented with complaints of acute abdominal pain, distension, absolute constipation, fever and vomiting for 2 days. He was treated for childhood tuberculosis with a nine month regimen of antitubercular drugs. He was asymptomatic thereafter. At admission the patient was febrile, tachycardic, with a distended, rigid and silent abdomen and was in shock. The total count was 12,600/cu mm, serum sodium was 134 meq/l, serum potassium 4.0 meq/l, BUN was 42 mg/dl and creatinine 1.8 mg/dl, and ESR was 110mm/hr. Erect plain radiographs of the chest and abdomen revealed free air under both domes of diaphragm. The patient was taken for emergency exploratory laparotomy. At laparotomy there was about one litre of feculent fluid in the peritoneal cavity. A perforation approximately 2 mm in diameter was seen on tip of Meckel’s diverticulum (adjacent a small daughter diverticulum) along with another synchronous perforation about 6 mm diameter, over the ileum, about one feet distal to the Meckel’s diverticulum. The operative findings were consistent with small bowel tuberculosis. About 2 feet of terminal ileum felt thickened, inflamed and edematous. The mesenteric lymph nodes were enlarged. A limited right hemicolectomy with ileo-ascendingcolon anastomosis, along with resection of 2 feet of thickened ileum including the Meckel’s diverticulum was done. The diverticulum measured 5 cm by 3 cm. The post operative period was uneventful and patient was discharged after 12 days after starting him on a course of antitubercular chemotherapy. Histopathological examination revealed tuberculosis in Meckel’s diverticulum, ileal perforation and mesenteric lymph node. The daughter diverticulum was made of only mucosa and was probably a false diverticulum.

## Discussion

Clinical features of Meckel’s diverticulitis are virtually indistinguishable from appendicitis, and operation is both diagnostic and therapeutic. It is not usually diagnosed before laparotomy for another condition or for one of its complications. Since the condition is not often diagnosed, it tends to be a forgotten entity until it perforates, bleeds or becomes gangrenous [3]. Majority of the cases of Meckel's diverticulum remain asymptomatic throughout life. About 4% of people with this anomaly develop complications, which include obstruction, haemorrhage, inflammation, perforation, neoplasia and Littre’s hernia [4]. In an excellent population-based study covering patient data over 42 years by Cullen et al, the lifetime risk of developing a complication that requires surgery was estimated to be 6.4% [5].

Multiple perforations of the bowel due to a single pathology are usually due to typhoid, tuberculosis or Non-Hodgkins lymphoma. Only very few cases of tubercular perforation of the Meckel’s diverticulum have been reported in literature [6, 7]. Intestinal tuberculosis is still a major problem in many regions of the world. A rising in incidence is noted in western countries due to immigration from Third World countries and human immunodeficiency virus infection [8]. Tuberculosis can involve the entire gastrointestinal tract, with common sites being the ileum and the ileo-caecal region. And in 90% of the cases, perforation is solitary, but multiple perforations occur in 10 - 40% of patients [9].

In this case, the possibility of Chron’s ileitis was also considered as a differential diagnosis. Free intestinal perforation may be the first sign of Chron’s disease in 23% of cases [10]. Perforation of the ileum due to chrons or tuberculosis has been described as spontaneous ileal perforations [11]. However in this case, histopathology was confirmatory of tuberculosis.

Surgical treatment of Meckel’s diverticulum includes either diverticulectomy or ileal resection. Simple diverticulectomy can suffice for asymptomatic diverticulum while, if there is induration of the base extending into the adjacent ileum, ileal resection is required. Absolute indications for resection are haemorrhage, intestinal obstruction, diverticulitis, and umbilico-ileal fistulas.

Optimal management of incidentally found Meckel’s diverticulum is controversial. Soltero et al [12], in their study opposed the practice of incidental diverticulectomy stating that the possibility of a Meckel’s diverticulum to become symptomatic in an adult patient is less than 2%, while morbidity rates of incidentally removed Meckel’s diverticulum were as high as 12%. However, Arnold et al reported 0% morbidity and mortality with removal of asymptomatic Meckel’s diverticulum [13].

In conclusion, tuberculous perforation of the Meckel’s diverticulum is a rare but noteworthy condition. Synchronous perforation of the ileum and Meckel’s diverticulum is also uncommon. Emergency laparotomy and resection of the affected small bowel segment including the diverticulum is the treatment of choice.
